# Larval diapause termination in the bamboo borer, *Omphisa fuscidentalis*

**DOI:** 10.1371/journal.pone.0174919

**Published:** 2017-04-03

**Authors:** Suphawan Suang, Manaporn Manaboon, Tippawan Singtripop, Kiyoshi Hiruma, Yu Kaneko, Pimonrat Tiansawat, Peter Neumann, Panuwan Chantawannakul

**Affiliations:** 1 Endocrinology Research Laboratory, Department of Biology, Faculty of Science, Chiang Mai University, Chiang Mai, Thailand; 2 Faculty of Agriculture and Life Sciences, Hirosaki University, Hirosaki, Japan; 3 Bee Protection Laboratory, Department of Biology, Faculty of Science, Chiang Mai University, Chiang Mai, Thailand; 4 Institute of Bee Health, Vetsuisse Faculty, University of Bern, Bern, Switzerland; 5 Agroscope, Swiss Bee Research Centre, Bern, Switzerland; Biocenter, Universität Würzburg, GERMANY

## Abstract

In insects, juvenile hormone (JH) and 20-hydroxyecdysone (20E) regulate larval growth and molting. However, little is known about how this cooperative control is terminating larval diapause especially in the bamboo borer, *Omphisa fuscidentalis*. In both *in vivo* and *in vitro* experiments, we here measured the expression levels of genes which were affected by juvenile hormone analogue (JHA: S-methoprene) and 20-hydroxyecdysone (20E) in diapausing *O*. *fuscidentalis* larvae. Corresponding mRNA expression changes in the subesophageal ganglion (SG) and prothoracic gland (PG) were evaluated using qRT-PCR. The data showed similar response patterns of JH receptor gene (*OfMet*), diapause hormone gene (*OfDH-PBAN*), ecdysone receptor genes (*OfEcR-A* and *OfEcR-B1*) and ecdysone inducible genes (*OfBr-C*, *OfE75A*, *OfE75B*, *OfE75C* and *OfHR3*). JHA induced the expressions of *OfMet* and *OfDH-PBAN* in both SG and PG, whereas ecdysone receptor genes and ecdysone inducible genes were induced by JHA only in PG. For 20E treatment group, expressions of ecdysone receptor genes and ecdysone inducible genes in both SG and PG were increased by 20E injection. In addition, the *in vitro* experiments showed that *OfMet* and *OfDH-PBAN* were up-regulated by JHA alone, but ecdysone receptor genes and ecdysone inducible genes were up-regulated by JHA and 20E. However, *OfMet* and *OfDH-PBAN* in the SG was expressed faster than *OfMet* and *OfDH-PBAN* in the PG and the expression of ecdysone receptor genes and ecdysone inducible genes induced by JHA was much later than observed for 20E. These results indicate that JHA might stimulate the PG indirectly via factors (*OfMet* and *OfDH-PBAN*) in the SG, which might be a regulatory mechanism for larval diapause termination in *O*. *fuscidentalis*.

## Introduction

Insect molting and metamorphosis are regulated by the interplay of two hormones, 20-hydroxyecdysone (20E) and juvenile hormone (JH) [1]. In insects, JH is generally synthesized by the *corpora allata* (CA), endocrine glands and contributes to the maintenance of larval growth. The 20E is transformed from ecdysone, produced in the prothoracic glands (PG) and triggers larval molting and metamorphosis with the larval-pupal transition [2]. The molting process is initiated in the brain, where neurosecretory cells release the neuropeptide prothoracicotropic hormone (PTTH) in response to neural, hormonal, or environmental factors [3]. PTTH stimulates PG to secrete molting hormone (ecdysteroids) [4]. The primary function of molting hormone is to stimulate the epithelial cells in the cuticle to begin the synthesis of a new exoskeleton [5]. In immature insects, JH inhibits the genes that promote development of adult characteristics (e.g. wings, reproductive organs, and external genitalia), causing the insect to remain "immature" (nymph or larva) [3, 6]. The CA becomes atrophied (shrink) during the last larval or nymphal instar and stops producing the JH [7]. This releases inhibition on development of adult structures and causes the insect to molt into an adult (hemimetabolous) or a pupa (holometabolous) [8]. Moreover, JH and 20E are intimately involved in regulating diapause. These hormones, together with diapause hormone (DH), a unique neuropeptide which is secreted from the subesophageal ganglion (SG), regulates the embryonic diapause of the commercial silkworm, terminates pupal diapause in *Helicoverpa* spp and regulates female reproductive maturation in adult insects [9–12]. The molecular mechanisms underlying the actions of 20E was firstly elucidated in the fruit fly (*Drosophila melanogaster*) and later confirmed in other insects [13]. Circulating 20-hydroxyecdysone then induces a systemic genetic response in multiple tissues by binding to a heterodimeric receptor comprised of the ecdysone receptor (EcR) and ultraspiracle (USP), both of which are transcription factors belonging to the nuclear receptor superfamily [14]. The liganded 20E-EcR-USP complex binds to ecdysone response elements (EcREs) located in the promoter regions of target genes and directly activates the transcription of the early ecdysone-inducible genes such as E74, E75 and Broad-Complex (BR-C), most of which encode transcription factors [15]. The products of early genes then activate the expression of early-late genes such as hormone receptor 3 (HR3). HR3 in turn inhibits E74, E75 and BR-C, permitting a developmental switch [16].

The mechanism mediating the JH response is a fascinating question. Recently, some studies have reported that JH could induce the transcription of a large number of genes *in vivo* or *in vitro* [17–19], and specific JH-response elements (JHREs) in some JH-regulated genes have been identified [20]. However, little is known about the crosstalk between JH and 20E in insect larvae. The molecular mechanisms underlying the actions of JH remain largely unknown. Recent studies show that methoprene-tolerant (Met), a basic-helix-loop-helix Per-Arnt-Sim (bHLH-PAS) family has been identified as the JH receptor [21]. JH binding to Met induces the heterodimerization of Met with steroid receptor co-activator (SRC) (also called Taiman or FISC) [21, 22]. The JH/Met/SRC complex binds to JH response elements (JHREs) located in the promoter regions of the target genes and induces their transcription. In the red flour beetle, *Tribolium castaneum*, Met plays a similar key role in JH action during the larval-pupal metamorphosis and precocious development of adult structures, indicating that Met is involved in antimetamorphic JH signaling [23, 24].

In holometabolous insects, Br-C are key players in the JH regulation of metamorphosis and is a transcription factor that directs pupal development [25]. In the larval stage, Met inhibits expression of BR-C that promotes pupal development [26]. The BR-C expressed specifically during larval-pupal metamorphosis under the control of 20E and JH is necessary for metamorphosis [27–29]. The role of BR-C in the larval-pupal transition has been studied in *D*. *melanogaster* [28], *B*. *mori* [30, 31], and *T*. *castaneum* [24, 32].

Larval diapause is generally known as an arrested period of post-embryonic development characterized by a major shutdown in metabolic activities and is under the control of hormonal interactions [33]. During larval diapause, a high level of JH in the hemolymph suppresses the activation of brain-prothoracic gland axis, preventing the release of ecdysteroids for larval growth and pupation [9, 34]. In the absence of ecdysteroids from the PG, the larva fails to initiate the next molt. The failure of the brain to release ecdysteroids can usually be directly attributed to the brain’s failure to release the PTTH [35]. While a low level or an absence of JH at the end of the larval or pupal stages allows 20E to promote metamorphosis [6]. In the southwestern corn borer, *Diatraea grandiosella*, the JH titer remains elevated throughout diapause and the diapause can be terminated only when the JH titer drops [36]. In some other species such as the European corn borer, *Ostrinia nubilalis*, the JH titer is high in early diapause but then declines and remains low throughout the remainder of diapause [37].

The bamboo borer (*Omphisa fuscidentalis*), a univoltine lepidopteran insect, is found in Northern Thailand, Laos and Myanmar [38]. The fifth instar larvae enter diapause and remain inside the internode of bamboo culm for nine months, from September until the following June. During the long larval diapause in *O*. *fuscidentalis*, the ecdysteroid titer in the hemolymph was found to be very low [38]. Topical application of juvenile hormone analogue (JHA) to diapausing *O*. *fuscidentalis* larvae induced pupation by increasing the ecdysteroid titer in hemolymph [39]. Previous studies have shown that, despite brain removal, pupation can be induced with JHA treatment in diapausing *O*. *fuscidentalis* larvae, indicating that the brain is not the primary target of the JHA [39]. When diapausing larvae of *O*. *fuscidentalis* were treated with JHA, the secretory activity of PG was increased at 10 days after JHA treatment [39]. When PG from JHA-treated larvae were dissected and transplanted to another non-treated larvae, the hemolymph ecdysteroid titer in those larvae exhibited a small increase within 16–18 days after transplantation [40]. This indicates that JHA does not activate the ecdysteroid biosynthesis in PG directly, but may stimulate through other tissues. In addition, recent studies have shown that treatment with exogenous diapause hormone (DH) could break the larval diapause of *O*. *fuscidentalis* by increasing levels of ecdysteroid in the hemolymph (Subta, P., unpublished work), correlated with diapause hormone gene (*OfDH-PBAN*) mRNA level during the development of *O*. *fuscidentalis* [41], suggesting that *OfDH-PBAN* transcript may be involved in regulating larval diapause termination. Although the effect of JHA on the expression of *DH-PBAN* genes has not been demonstrated in other insect’s tissues, our preliminary experiment showed that the expression of *OfDH-PBAN* mRNA in SG was much higher than PG (data not shown). This result indicated that *DH-PBAN* gene was synthesized primarily in SG [42]. Hence, we assumed that JHA might activate the DH-PBAN biosynthesis in SG directly. Consequently, it may be possible that JHA directly stimulates the SG by activating SG to synthesize DH-PBAN, which is then released into the hemolymph and acts on the PG in order to release ecdysone, thereby leading to larval diapause termination. However, the mechanism for breaking larval diapause by JHA is still unknown. Therefore, in this study we examined the effects of JHA and 20E on the expressions of *OfMet*, *OfDH-PBAN*, ecdysone receptor genes (*OfEcR-A* and *OfEcR-B1*) and ecdysone inducible genes (*OfBr-C*, *OfE75A*, *OfE75B*, *OfE75C* and *OfHR3*) in SG and PG both *in vivo* and *in vitr*o. The knowledge of expression pattern of *OfMet*, *OfDH-PBAN*, ecdysone receptor genes and ecdysone inducible genes may help us to better understand the mechanisms of larval diapause termination in *O*. *fuscidentalis*.

## Materials and methods

### Sampling

From November to December 2014, larvae of *O*. *fuscidentalis* were sampled from bamboo forests in the Maewang District, Chiang Mai Province, Thailand (No specific permissions were required for the bamboo forest where the bamboo borers were collected). In addition, the field studies in this research did not involve endangered or protected species. No specific permissions were required for the bamboo forest where the bamboo borers were collected. Diapause in this *O*. *fuscidentalis* population usually lasts from September to June [38]. Therefore, the sampled larvae are most likely representative of the diapause stage and were kept in containers [12 x 14 x 8 cm] lined with sheets of wet paper towel, which were maintained in darkness at 25°C and 95% RH following Singtripop *et al*. (1999).

### Hormones

The doses of JHA and 20E (0.5 μg/larva) were able to induce pupation according to the established works by Singtripop *et al*. (2000, 2002). The selected dose was used as a critical dose in all experiments. The JHA (S-methoprene, >95% stereochemically pure; SDS Biotech, Japan) was dissolved in acetone (final concentration 5 mg mL^-1^) and stored at -35°C as a stock solution. The stock solution was diluted to 0.5 μg per 5 μL with acetone, and a 5 μL aliquot was applied to the dorsal abdomen of individual diapausing larvae using a 50 μL microsyringe (N = 700) [39]. Individuals in the controls were treated with 5 μL acetone only (N = 700). As previously reported, the larvae (designated as G0) became motionless and their epidermis turned brown and formed a hard, pigmented cuticle after JHA treatment. The physiological ages of the larvae were categorized into six stages, from G0 to G5, based on body coloration and deposition of pigmented pupal cuticle [39].

The 20-hydroxyecdysone (20E, Sigma, St. Louis, Missouri), was dissolved in distilled water (DW) at a concentration of 1 mg mL^-1^ and stored at -35°C until use. The stock solution was diluted to 0.5 μg per 5 μL in DW, and diapausing larvae were injected with a 5 μL aliquot through the second proleg (N = 700) [43]. Control larvae were injected with 5 μL DW (N = 700).

### RNA isolation and cDNA synthesis

Total RNA was isolated from SG and PG using RiboZol RNA Extraction reagent (Amresco, Solon, Ohio) according to the manufacturer’s instructions. Before cDNA synthesis, the RNA was treated with RNase-free DNaseI (Fermentas, Vilnius, Lithuania) to eliminate contaminating DNA. A quantity of 1 μg of total RNA was used to generate first-strand cDNA with an oligo-dT primer and M-MuLV-Reverse Transcriptase (Fermentas).

### Effects of JHA and 20E on *OfMet*, *OfDH-PBAN*, ecdysone receptor genes and ecdysone inducible genes expression *in vitro*

A diluted stock solution of JHA (0.02 μg μL^-1^) was prepared in EtOH. The solution was evaporated in air and resuspended in Grace’s insect cell culture medium (Gibco Invitrogen Corporation, Grand Island, New York). All equipment used in the JHA experiments was coated with 1% solution of polyethylene glycol. For the 20E experiments, the stock solution (0.02 μg μL^-1^) was prepared in DW. Both JHA and 20E stock solutions were serially diluted in culture medium to the appropriate working concentrations. In brief, the diapausing larvae were anesthetized on ice for 30 min. The SG and PG were dissected and rinsed 5 times in ice-cold Ringer’s solution (130 mM NaCl, 4.7 mM KCl and 1.9 mM CaCl_2_). The tissue was rinsed again in culture medium five times. The hormones were then added to 50 μL medium containing 1 lobe of SG and PG in 96-well culture plates (Sero-Wel, Bibby Sterilin, U.K.) and were incubated at 25°C with gentle shaking. For the controls, equal volumes of the medium without JHA or 20E were added. Total RNAs from SG and PG in both hormone treatments and control experiments were extracted at the same time points after incubation.

### Quantitative real-time PCR (qRT-PCR)

The primers used for amplifying cDNA fragment are listed in Table 1. The expression level of p49, the ortholog of ribosomal protein L3 (*OfRpL3*), is the most stable and constant in *B*. *mori* nervous system [44]. For normalization of qRT-PCR, we tested *OfRpL3* expression, and found that *RpL3* showed the constant expressions in PG and SG of *O*. *fuscidentalis* [40]. Therefore, *OfRpL3* mRNA was used as an endogenous control. Quantitative real-time PCR was conducted using the SensiFAST^TM^ SYBR^®^ No-Rox Kit (Bioline, U.K.) and an iCycler iQ5™ Real-Time PCR Detection System (Bio-Rad, Hercules, California). One reaction contained 1 μL of template cDNA sample and 0.2 μM primers in a final reaction volume of 20 μL. The thermal cycling parameters were 95°C for 2 min, followed by 40 cycles at 95°C for 5 sec, 60°C for 10 sec, and 72°C for 20 sec. After qRT-PCR, the absence of undesired by-products was confirmed by an automated melting curve analysis and agarose gel electrophoresis (1.5% w/v) of the PCR product. Also, amplified PCR products were purified by using a GeneJET Miniperp Kit (Fermentas) and DNA sequencing was performed by an ABI PRISM Bigdye Terminator (version 3.1) cycle Sequencing Kit (Applied Biosystems, Foster City, California) and automated DNA sequencer (ABI PRISM 3100 Genetic Analyzer, Applied Biosystems). The amplification efficiency for both the reference and target genes was analyzed. The relative expression levels of *OfMet*, *OfDH-PBAN*, ecdysone receptor genes (*OfEcR-A* and *OfEcR-B1*) and ecdysone inducible genes (*OfBr-C*, *OfE75A*, *OfE75B*, *OfE75C* and *OfHR3*) from samples with different treatments were estimated by a comparative C_T_ method (ΔΔC_T_) for relative quantitation of gene expression. The C_T_ (cycle threshold) is defined as the number of cycles required for the fluorescent signal to cross the background level. The dynamic range of the target genes and normalizer (*OfRpL3*) were determined. After normalization with *OfRpL3* [i.e., ΔC_T_ = C_T (*OfMet*, *OfDH-PBAN*, ecdysone receptor genes or ecdysone inducible genes)_—C_T (*OfRpL3*)_], the ΔC_T_ value of the treatment group was compared with that of the control group known as the calibrator [45] (i.e., ΔΔC_T_). All results of qRT-PCR were analyzed using Pfaffl’s mathematical model [46].

**Table 1:** Forward and reverse primers used in the qRT-PCR analyses.

Gene name	Forward primer	Reverse primer	Accession no.
***Met***	TTTGTAGGTGTCGTGCGTGT	CGCTCGTGTGTCATGTATCC	KT223005
***DH-PBAN***	TATGAGAGTCGAGCTGATGAC	GTGACGGAAAGCTTCTCCGG	JX195641
***EcR-A***	GTGAAATCGTGAGGCCGTAT	ACACTTCACTGGAGGCGTTT	EF667890
***EcR-B1***	TGCGTGGATTGTGTTTTGTT	CACTTTTTCCCCGCACTAAA	EF667891
***Br-C***	GCGCATAGAGTGGTCCTTTC	GGTTTTCAGGAACGAGGACA	KT223007
***E75A***	CATTACGGTGTGCATTCCTG	GACAGCATCTCTGCTCATGC	KT224348
***E75B***	CGGTGCTAGTGAGCATGTTG	AGGATGGAGCACTGCTGATT	KT224349
***E75C***	CCGGATCTAGAGTTCGATGG	AGGATGGAGCACTGCTGATT	KT223004
***HR3***	CCAACGCAGTCCGATGGATA	GCCCATCCCACTCAAGTTCA	KT223006
***RpL3***	TCTACCCCAAGAAGAGGTCTCG	ACGACAGTCCTCAGACATGTGC	EF453378

### Statistical analysis

In this study, there were two main parts of the experiments, including *in vivo* and *in vitro* experiments. Both datasets were analyzed separately using SPSS version 17.0 (SPSS, Inc.). For the *in vivo* datasets, the effects of JHA and 20E on *OfMet*, *OfDH-PBAN*, ecdysone receptor genes and ecdysone inducible genes expression were examined separately for SG and PG. To determine whether the relative expressions differ among larval and pupal development stages, we used the one-way analysis of variance (ANOVA) to analyze each expression. The means of relative expression levels from three replicates was used as a dependent variable and the stages of larval and pupal development was used as an independent variable. For the JHA dataset, the stages of larval development were categorically independent variable with 11 levels—day 0, 2, 4, 6, 8, 10, 12 after the treatment and four pupal stages (G0-G3). For the 20E dataset, the larvae went into pupal stages sooner than those in the JHA treatment. Therefore, the stages of larval development in the 20E treatment contained 10 levels—day 0, 2, 4, 6, 8, 10 after the treatment and four pupal stages (G0-G3). The normality of dependent variable residuals and the homogeneity of variances of the data were checked to ensure that the assumptions of ANOVA were met. When the results of ANOVA showed that there were differences in the relative expression levels among the stages of larval and pupal development, a Tukey’s HSD test was performed to obtain the information on which means are significantly different from each other at a significance level of 0.01. Data are presented as the mean ± SE.

For the *in vitro* dataset, in addition to JHA and 20E treatment, the data on combined effects of JHA+20E were also analyzed. The means of relative expression levels from three replicates was used as a dependent variable and the time after the treatment application was used as a categorically independent variable with 9 levels—0, 0.5, 1, 4, 8, 12, 16, 20 and 24 hours after the treatment application. The analyses were performed in the same fashion as the *in vivo* datasets.

## Results

### *In vivo* effects of JHA on *OfMet*, *OfDH-PBAN*, ecdysone receptor genes and ecdysone inducible genes expression in both SG and PG

**Fig 1:**
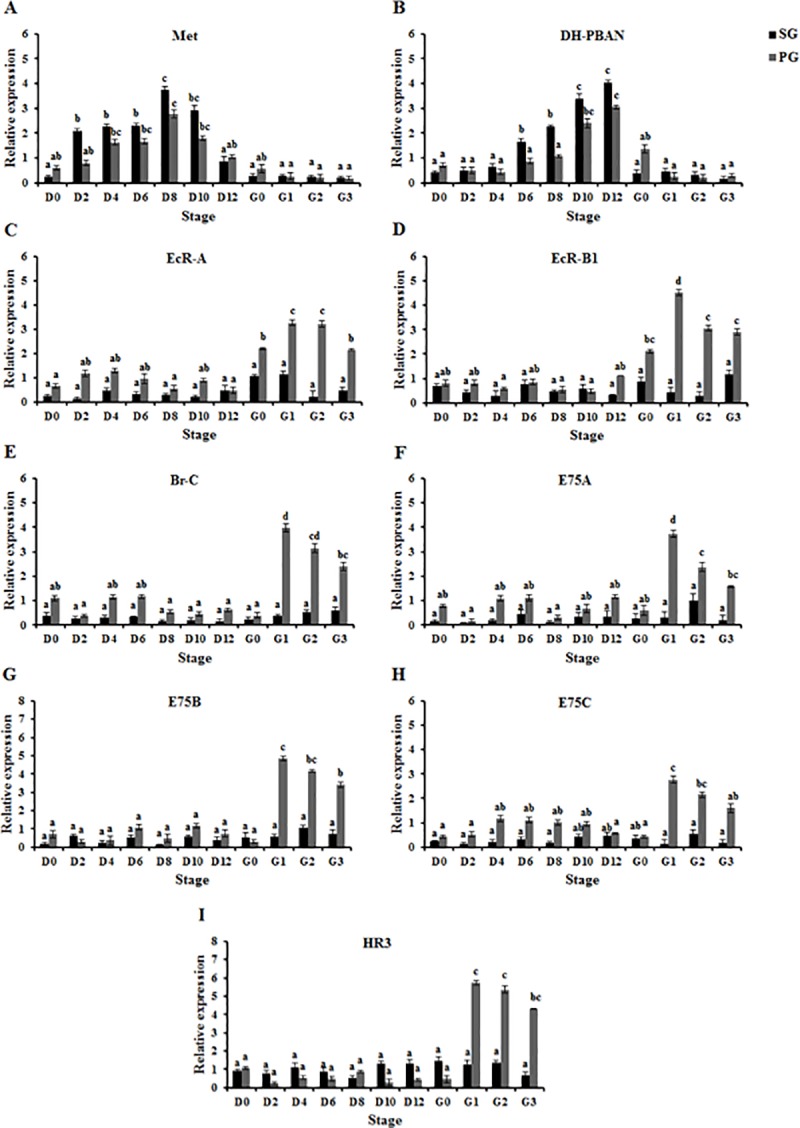
JHA-mediated induction of *OfMet*, *OfDH-PBAN*, ecdysone receptor genes and ecdysone inducible genes expression in SG and PG of diapausing *Omphisa fuscidentalis* larvae. Larvae were treated with 0.5 μg JHA, and *OfMet* (A), *OfDH-PBAN* (B), *OfEcR-A* (C), *OfEcR-B1* (D), *OfBr-C* (E), *OfE75A* (F), *OfE75B* (G), *OfE75C* (H) and *OfHR3* (I) mRNA levels were determined from the total RNA pool after treatment; D0-D12, 0–12 days after treatment, G0-G3, stages in pupal development. Means and standard deviations are shown. Bars sharing the same letter are not significantly different according to Tukey’s HSD test.

We induced pupation by treating diapausing larvae with JHA (the treated diapausing larvae entered pupation stage at Day 12) and examined the effect of JHA on expression levels of *OfMet*, *OfDH-PBAN*, ecdysone receptor genes and ecdysone inducible genes in both SG and PG (Fig 1). After the treatment of 0.5 μg JHA, *OfMet* mRNA levels in the SG were low on day 0, while levels of *OfMet* mRNA in the PG were low until day 2. Thereafter, it then significantly increased and remained until reaching its maximum level on day 8 (ANOVA, *P* < 0.01), however it later decreased on day 10 and abruptly dropped to a minimum level until G3 stage (Fig 1A). In addition, *OfDH-PBAN* mRNA levels in the SG were low from days 0 to 4, and then gradually increased until reaching a maximum level on day 12, while *OfDH-PBAN* mRNA levels in the PG were low from days 0 to 8, and then increased dramatically until reaching a peak on day 12 (ANOVA, *P* < 0.01). Expression of *OfDH-PBAN* in both SG and PG then abruptly dropped to a low level at G0-G3 stage (Fig 1B). In the SG, the expression level of ecdysone receptor genes and ecdysone inducible genes was expressed at a very low level between day 0 to G3 stage and was not significant when compared with controls (Fig 1C–1I). By contrast, the expression level of ecdysone receptor genes in PG was low from days 0 to 12, and then increased dramatically to a high level at G0-G3 stage (ANOVA, *P* < 0.01) (Fig 1C and 1D). In addition, the expression levels of ecdysone inducible genes in the PG were low between day 0 and G0 stage, then rapidly increased to the maximal value at the G1-G3 stage (ANOVA, *P* < 0.01) (Fig 1E–1I).

These results indicated that JHA significantly increased expression levels of *OfMet* and *OfDH-PBAN* in SG and PG. JHA also induced the expression levels of ecdysone receptor genes and ecdysone inducible genes in PG during pupal stage (ANOVA, *P* < 0.01).

### *In vivo* effects of 20E on *OfMet*, *OfDH-PBAN*, ecdysone receptor genes and ecdysone inducible genes expression in both SG and PG

**Fig 2:**
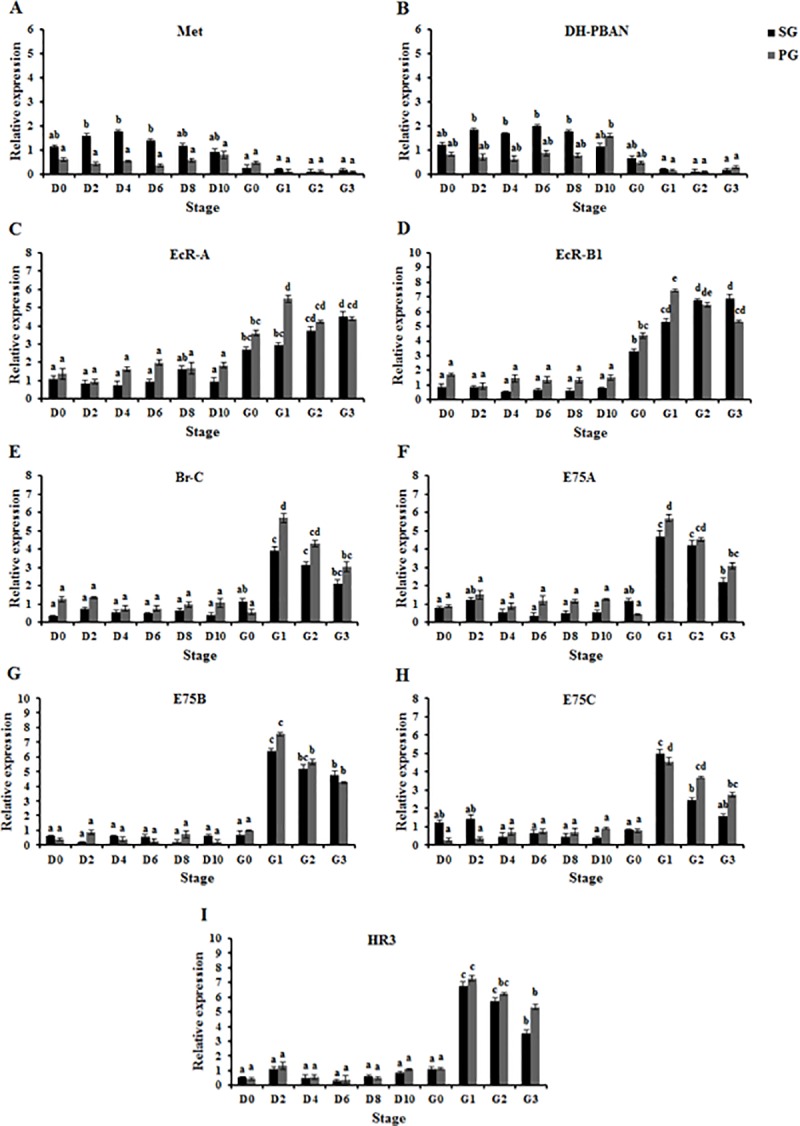
20E-mediated induction of *OfMet*, *OfDH-PBAN*, ecdysone receptor genes and ecdysone inducible genes expression in SG and PG of diapausing *Omphisa fuscidentalis* larvae. Larvae were injected with 0.5 μg of 20E, and *OfMet* (A), *OfDH-PBAN* (B), *OfEcR-A* (C), *OfEcR-B1* (D), *OfBr-C* (E), *OfE75A* (F), *OfE75B* (G), *OfE75C* (H) and *OfHR3* (I) mRNA levels were determined from the total RNA pool after treatment; D0-D10, 0–10 days after treatment. Means and standard deviations are shown. Bars sharing the same letter are not significantly different according to Tukey’s HSD test.

We also examined the effects of 20E on the expression levels of *OfMet*, *OfDH-PBAN*, ecdysone receptor genes and ecdysone inducible genes in both SG and PG (Fig 2). In larvae injected with 0.5 μg 20E which turned browned and entered pupal stage at Day 10 after treatment, the expression levels of *OfMet* and *OfDH-PBAN* in both SG and PG were low from day 0 to G3 stage and they were not significantly different when compared with controls (Fig 2A and 2B). Moreover, the expression levels of ecdysone receptor genes in both SG and PG were low from days 0 to 10, and then increased gradually until reaching a maximum level at G1-G3 stage (ANOVA, *P* < 0.01) (Fig 2C and 2D). Furthermore, the expression levels of ecdysone inducible genes were low between days 0 and G0 stage, then increased dramatically and peaked at G1 stage, followed by a decline at G2 and G3 stage (ANOVA, *P* < 0.01) (Fig 2E–2I).

### *In vitro* effects of JHA and 20E on *OfMet*, *OfDH-PBAN*, ecdysone receptor genes and ecdysone inducible genes expression in the SG

To understand how hormonal mechanism on termination of larval diapause is regulated by JHA, the SG was cultured in Grace’s medium containing both JHA at 0.1 μg/50 μl and 20E at 0.1 μg/50 μl for different continuous time exposures. The expression patterns of *OfMet*, *OfDH-PBAN*, ecdysone receptor genes and ecdysone inducible genes were examined under these experimental conditions.

**Fig 3:**
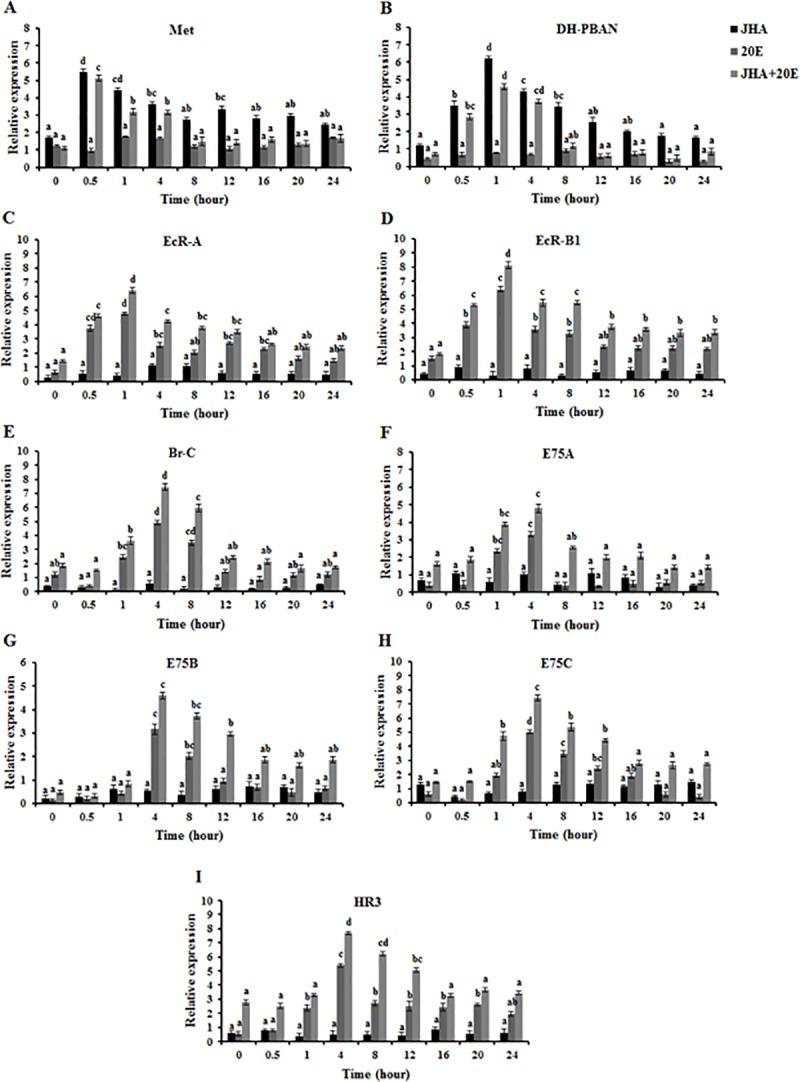
Induction of JHA and 20E on *OfMet*, *OfDH-PBAN*, ecdysone receptor genes and ecdysone inducible genes expression in the SG of diapausing *Omphisa fuscidentalis* larvae *in vitro*. Time course analyses of *OfMet* (A), *OfDH-PBAN* (B), *OfEcR-A* (C), *OfEcR-B1* (D), *OfBr-C* (E), *OfE75A* (F), *OfE75B* (G), *OfE75C* (H) and *OfHR3* (I) mRNA after treatment with 0.1 μg/50 μl of JHA alone, 0.1 μg/50 μl of 20E alone or 0.1 μg/50 μl of JHA and 20E together determined at 0–24 h. Means and standard deviations are shown. Bars sharing the same letter are not significantly different according to Tukey’s HSD test.

We remarked that in the presence of JHA alone at 0.1 μg/50 μl, the *OfMet* mRNA levels were low at 0 h, and then rapidly increased to the maximal value at 0.5 h (ANOVA, *P* < 0.01), followed by a decline from 1 to 24 h (Fig 3A), while the expression level of *OfDH-PBAN* was low at 0 h, reached a maximum at 1 h and then decreased at 4–24 h (ANOVA, *P* < 0.01) (Fig 3B). By contrast, JHA did not significantly up-regulate ecdysone receptor genes and ecdysone inducible genes expression in SG (Fig 3C–3I). In the presence of 20E alone, *OfMet* and *OfDH-PBAN* mRNA levels were constitutively expressed at a low level over time (ANOVA, *P* > 0.01) (Fig 3A and 3B) whereas the expression level of ecdysone receptor genes was low at 0 h, then dramatically increased at 0.5 h, reached the highest level at 1 h (ANOVA, *P* < 0.01), followed by a large decrease at 4 h and remained steady at a low level until 24 h (Fig 3C and 3D). The relative expressions of ecdysone inducible genes were low at 0–1 h, then increased dramatically to the maximal value at 4 h (ANOVA, *P* < 0.01) and decreased at 8–24 h after 20E application (Fig 3E–3I). These results indicated that JHA induced the expressions of *OfMet* and *OfDH-PBAN*, while 20E could only induce the expressions ecdysone receptor genes and ecdysone inducible genes in SG.

Moreover, we performed a time course experiment to determine how the presence of the two hormones (JHA+20E) together affected gene expressions as a function of time. When SG were co-cultured with 0.1 μg/50 μl of JHA and 20E, *OfMet* and *OfDH-PBAN* transcripts showed temporal patterns similar to those obtained in response to JHA (ANOVA, *P* < 0.01) (Fig 3A and 3B), while expression patterns of ecdysone receptor genes and ecdysone inducible genes were similar to those obtained in response to 20E (ANOVA, *P* < 0.01) (Fig 3E–3I).

### *In vitro* effects of JHA and 20E on *OfMet*, *OfDH-PBAN*, ecdysone receptor genes and ecdysone inducible genes expression in the PG

**Fig 4:**
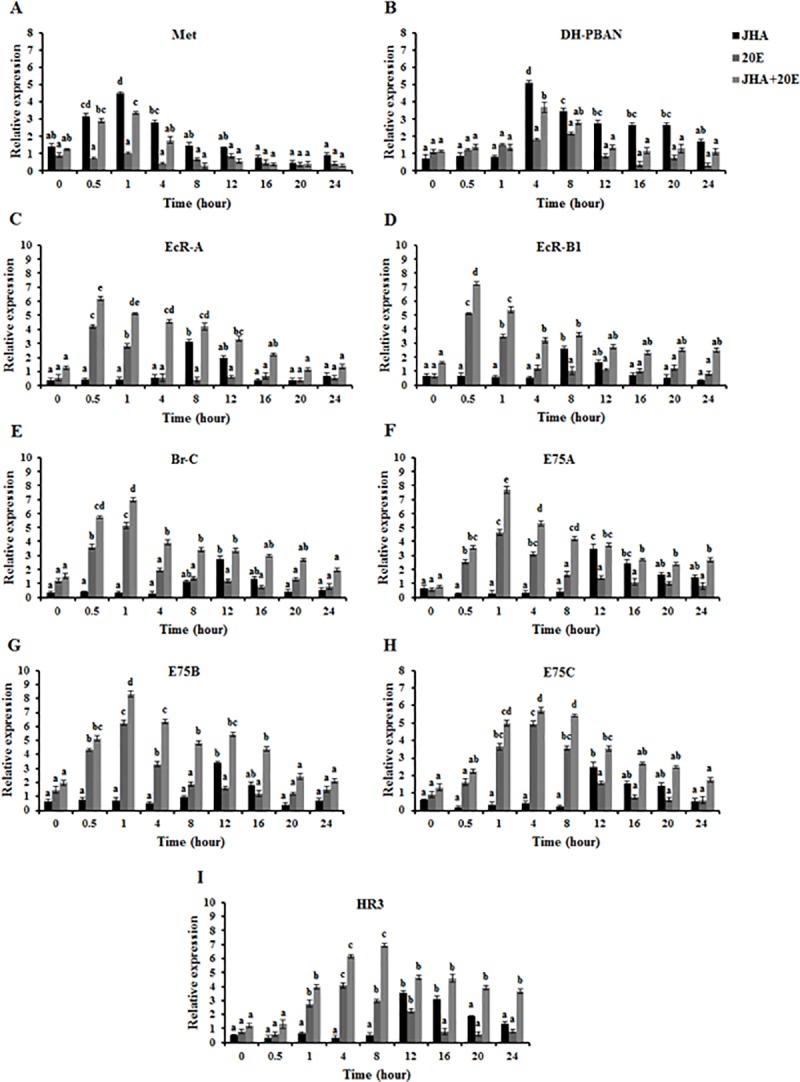
Induction of JHA and 20E on *OfMet*, ecdysone receptor genes and ecdysone inducible genes expression in the PG of diapausing *Omphisa fuscidentalis* larvae *in vitro*. Time course analyses of *OfMet* (A), *OfDH-PBAN* (B), *OfEcR-A* (C), *OfEcR-B1* (D), *OfBr-C* (E), *OfE75A* (F), *OfE75B* (G), *OfE75C* (H) and *OfHR3* (I) mRNA after treatment with 0.1 μg/50 μl of JHA alone, 0.1 μg/50 μl of 20E alone or 0.1 μg/50 μl of JHA and 20E together determined at 0–24 h. Means and standard deviations are shown. Bars sharing the same letter are not significantly different according to Tukey’s HSD test.

We also examined the effect of JHA and 20E on the changes of *OfMet*, *OfDH-PBAN*, ecdysone receptor gene and ecdysone inducible gene expressions in PG, the PG was incubated in Grace’s medium containing both 0.1 μg/50 μl of JHA and 0.1 μg/50 μl of 20E as a function of time. In the presence of JHA alone, the results showed that the relative expression of *OfMet* was low at 0 h, rapidly increased at 0.5 h, peaked at 1 h (ANOVA, *P* < 0.01) and then decreased at 4–24 h, while *OfDH-PBAN* was low between 0 and 1 h, then increased suddenly to a maximum level at 4 h (ANOVA, *P* < 0.01), followed by a decrease at 8 h and remained at low levels from 12–24 h (Fig 4A and 4B). Moreover, the expression level of ecdysone receptor genes was low from 0–4 h, and then increased dramatically to the maximal value at 8 h (ANOVA, *P* < 0.01), followed by a decline at 12–24 h (Fig 4C and 4D). In addition, the expression levels of ecdysone inducible genes were low from 0 to 8 h, increased rapidly to a maximum level at 12 h (ANOVA, *P* < 0.01), then declined and remained steady at a low level until 24 h after JHA addition (Fig 4E–4I). In the presence of 20E alone, *OfMet* and *OfDH-PBAN* mRNA levels remained at a low level at 0–24 h and were not significant when compared with the controls (Fig 4A and 4B). By contrast, the relative expression of ecdysone receptor genes was low at 0 h, then rapidly increased to a maximum level at 0.5 h (ANOVA, *P* < 0.01) and decreased at 1 h. Expression of ecdysone receptor genes then declined abruptly to low levels until 24 h (Fig 4C and 4D). The expression levels of *OfBr-C*, *OfE75A* and *OfE75B* were low at 0 h, and then increased dramatically until reaching a maximum level at 1 h (ANOVA, *P* < 0.01). Thereafter, it decreased at 4 h and then remained at low levels from 8–24 h (Fig 4E–4G). *OfE75C* and *OfHR3* mRNA levels gradually increased and peaked at 4 h (ANOVA, *P* < 0.01), followed by a decline at 8–24 h after the application of 20E (Fig 4H and 4I). In combination of both JHA and 20E for the time- course experiment, *OfMet* and *OfDH-PBAN* mRNA levels showed a similar pattern on those obtained in response to JHA alone (ANOVA, *P* < 0.01) (Fig 4A and 4B), while the expression levels of ecdysone receptor genes and ecdysone inducible genes showed a similar pattern on those obtained in response to both JHA and 20E (ANOVA, *P* < 0.01) (Fig 4C–4I).

## Discussion

In our study, both *in vivo* and *in vitro* experiments results displayed similar patterns of expressions of *OfMet*, *OfDH-PBAN*, ecdysone receptor genes and ecdysone inducible genes in SG and PG. Interestingly, JHA induced the expression of *OfMet* and *OfDH-PBAN* in SG and PG whereas 20E had no significant effect on expression of *OfMet* and *OfDH-PBAN* in both tissues, indicating that 20E might not be involved in the up-regulation of *OfMet* and *OfDH-PBAN* genes expression in SG and PG but both genes were directly up-regulated by JHA only. Our results of *OfMet* expression patterns in bamboo borer were similar to that of *BgMet* in whole body of *Blattella germanica* when treated with juvenile hormone [47]. Recently, Met has been proclaimed as the JH receptor [21, 48]. This correlates with our results in which the induction of *Met* was shown as a rapid response to JHA, indicating that JH itself may plays a crucial role in regulating the expression of its receptor [49]. In natural system, endogenous JH in insect larvae (e.g. *T*. *castaneum*, *D*. *melanogaster* and *B*. *mori*) maintain continuous expression of Krüppel homolog 1 (*Kr-h1*), which is an early JH-response gene via *Met*, and *Kr-h1* inhibits metamorphic changes [26, 50, 51]. However, a previous study has shown that exogenous JH mimic (JHM) could induce *Kr-h1* expression via *Met* in the pupal stage of *D*. *melanogaster* [50] and *T*. *castaneum* [26], which in turn causes induction of *Br-C* and formation of second pupae. Moreover, JH induced the expression of ecdysone inducible genes such as *Br-C* [25, 28], *E75* [52, 53] and *HR3* [54]. This coincides with our results in which JHA increased the expression levels of ecdysone receptor genes and ecdysone inducible genes only in PG. These suggest that *Met* and *Kr-h1* could be involved in mediating JH signals. Therefore, it is possible that JH affects the expression of 20E-related genes via *Met* and *Kr-h1* and *Met* plays a role in mediating termination process of larval diapause in *O*. *fuscidentalis*.

According to Suang *et al*. (2015), the expression of *DH-PBAN* was generally observed in neural tissues including the brain, SG, the thoracic ganglion and the abdominal ganglion. The highest expression was shown in SG. To study the signaling pathway of JHA on diapause termination in the bamboo borer, and involvement of PG as a source for the elevation of ecdysteroid, we focused on *DH-PBAN* expression in PG. Without stimulating PG with JHA, we could measure the expression levels of DH-PBAN in PG of natural larvae and pupae S1 Fig. However, DH-PBAN/hugin/pyrokinin is produced and released by neurons in the SG that innervate the corpora cardiaca in many insect species [55]. Similarly, in Lepidopteran insects, the PG is innervated by nerves from the SG [56, 57]. There have been, however, no reports of DH-PBAN being transcribed by PG cells themselves and there is also no evidence in any insect that neuropeptides can be derived from PG cells. We presume, from our results, that the detectable *DH-PBAN* mRNA may originate from terminals of the DH-PBAN neurons with somata in the SG. In addition, the DH-PBAN neurons may even innervate the PG in the bamboo borer. Thus, we would like to propose an alternative hypothesis that the *DH-PBAN* mRNA, originally expressed from the terminals of DH-PBAN SG neurons, might be present in the isolated PG samples.

To clarify the specificity of *OfDH-PBAN*, the specific primers for *OfDH-PBAN* were designed (according to Suang *et al*., 2015) and used to amplify the gene in PG. A single band was clearly evident in the gel after electrophoresis S2A Fig. Also the amplified product sequences showed 100% similarity to the *OfDH-PBAN* nucleotide sequence S2B Fig. Thus, it indicated that *OfDH-PBAN* was expressed in PG.

Not only *OfMet*, but *OfDH-PBAN* was also upregulated by JHA. It was clear that *OfMet* and *OfDH-PBAN* in the SG were expressed much faster than *OfMet* and *OfDH-PBAN* in PG, suggesting that one of the functions of JH in the termination of larval diapause could be to directly stimulate *OfMet* and *OfDH-PBAN* expressions in the SG. Nevertheless, the mechanism of DH to activate PG in releasing ecdysone, and then stimulate larvae to break the diapause is still unknown. Our preliminary experiment showed that exogenous DH could also break larval diapause of *O*. *fuscidentalis* by increasing levels of ecdysteroid in the hemolymph (Subta, P., unpublished work). This result was also in agreement with a previous study obtained from *Helicoverpa spp*. [58]. Moreover, Watanabe *et al*. (2007) found that the *Bombyx* diapause hormone receptor (BmDHR) was expressed in the PG, and the FXPRL-amide peptides were able to induce ecdysteroidogenesis in the PG through the activation of BmDHR [59]. Therefore, *OfDH-PBAN* genes might be one of the hemolymph factors to terminate larval diapause, after being synthesized from the SG and released into the hemolymph in *O*. *fuscidentalis*. According to our results, *OfDH-PBAN* mRNA was also expressed in PG. In this study, we focused on mRNA expression to display the correlation at transcription level between JHA, *DH-PBAN* and diapause termination in the bamboo borer. The effects of JHA on DH-PBAN at a protein expression level will need to be investigated to reaffirm the action of JHA and DH-PBAN.

In *O*. *fuscidentalis*, 20E is tightly involved in the termination of the larval diapause by increasing the ecdysteroid titer in the PG directly [40]. This correlates with our results, in which 20E increased the expression levels of ecdysone receptor genes and ecdysone inducible genes in both SG and PG. It has been also reported *in vitro* that the *EcR* genes were directly stimulated by 20E in the larvae of Lepidopterans such as *M*. *sexta* [60], *P*. *interpunctella* [54], and *B*. *mori* [61]. Furthermore, it was found that exogenous 20E induced the expressions of *EcR* genes [29, 62, 63] and ecdysone inducible genes such as *Br-C* [60, 64, 65], *E75* [54, 66–69] and *HR3* [70, 71]. Meanwhile, JHA had no significant effect on an increase of the expression levels of ecdysone receptor genes and ecdysone inducible genes in SG. This result of the expression level of ecdysone receptor genes was in agreement with that obtained on *EcR* homologous gene in the *M*. *sexta* epidermis which JH prevented the 20E-induced metamorphic switching by regulating the induction of *EcR* by 20E [72]. The effect of JHA on *OfE75* expression was similar to the previous reports for *D*. *melanogaster* [19] and *M*. *sexta* [73, 74]. After brain removal, *O*. *fuscidentalis* larvae were treated with 1 μg JHA, the ecdysteroid titer increased in the hemolymph. This indicated that the brain was not the target tissue for JHA [39]. In addition, it seemed that JHA was indirectly stimulating the PG because it activated the ecdysteroid biosynthesis [40]. Our results suggest that JHA might play a role in up-regulating the expression of ecdysone receptor genes and ecdysone inducible genes in PG via *OfMet* and *OfDH-PBAN* in SG and then acting on PG to release ecdysone. Thus, the ecdysteroids produced by the PG might have up-regulated the *OfEcR* genes in prothoracic gland cells, which in turn causes induction of ecdysone inducible genes and termination of larval diapause in *O*. *fuscidentalis*.

In addition, the simultaneous treatment with JHA and 20E displayed combined effects of JHA and 20E. The increase in the expressions of ecdysone receptor genes and ecdysone inducible genes in PG incubated with both JHA and 20E was higher than PG incubated in medium containing either JHA or 20E. This observation suggested that the presence of both hormones increased expression levels of these genes in PG. However, the expression levels of ecdysone receptor genes and ecdysone inducible genes were induced by JHA much later than observed for 20E *in vitro*. These results indicate that JHA did not directly up-regulate the expressions of ecdysone receptor genes and ecdysone inducible genes in PG, but JHA could stimulate ecdysone synthesis in PG via *OfMet* and *OfDH-PBAN* in SG, which then could feed back on the glands to induce the synthesis of these genes within the gland.

In addition, for *in vivo* experiments, the expression levels of *OfMet* and *OfDH-PBAN* before entering the pupal stage and the expression levels of ecdysone receptor genes and ecdysone inducible genes in the pupal stage may be associated with an increment of JH and 20E titer in the hemolymph. In *O*. *fuscidentalis*, the hemolymph ecdysteroid concentration is low during larval diapause and increases prior to the pupal stage, thereby presumably stimulating pupal metamorphosis [38]. However, JH titer during development of *O*. *fuscidentalis* has not been determined. A previous study has shown that the expression level of juvenile hormone binding protein (JHBP) encoding gene in the fat body was high in the diapause period and low from the late-diapause until pupation [75]. Change in the JH titer in the hemolymph toward diapause termination is still to be determined.

Amongst insects, *O*. *fuscidentalis* has the longest larval diapause period to adapt themselves to the natural habitats [38]. Juvenile hormones induce pupation in the diapausing larvae of *O*. *fuscidentalis* while a high level of juvenile hormone maintains larval stage in other lepidopteran insects. The finding in this study may help us to understand the relationship between JH and 20E on termination of larval diapause in *O*. *fuscidentalis*, which JH might play a crucial role in regulating ecdysteroid signaling pathway in this mechanism. Further studies to analyze expression level of the enzymes, which are related with ecdysteroid production are needed to elucidate the hormonal regulation of PG activity and of the enzymes which are related with ecdysteroid production. of the enzymes which are related with ecdysteroid production. of the enzymes which are related with ecdysteroid production. the unique regulatory mechanism of *O*. *fuscidentalis* larval diapause.

## Supporting information

S1 Fig Developmental changes of *OfDH-PBAN* mRNA in prothoracic gland during diapause (October to April) and post-diapause (pupation) which were measured by a quantitative real time polymerase chain reaction. The results are expressed as the relative expression after normalization against endogenous ribosomal protein mRNA (*OfRpL3*). Expression is relative to the gene expression in diapausing larvae from October (assigned a value of 1). Each value is the mean ± SEM of three independent experiments. Means with different letters indicate a significantly difference (ANOVA: n = 3, P < 0.05)
(PDF)

S2 Fig The specificity of the DH-PBAN primers. (A) PCR product of *OfDH-PBAN* mRNA by using primers OfDH-SPF and OfDH-SPR. M, marker for DNA molecular weight. (B) Nucleotide sequence and the deduced amino acid sequence of the PCR products.
(PDF)
