# Supplementary figures and images for: Larval diapause termination in the bamboo borer, Omphisa fuscidentalis
Source: PLoS One. 2017 Apr 3;12(4):e0174919. doi: 10.1371/journal.pone.0174919 (PMC5378396; doi:10.1371/journal.pone.0174919)

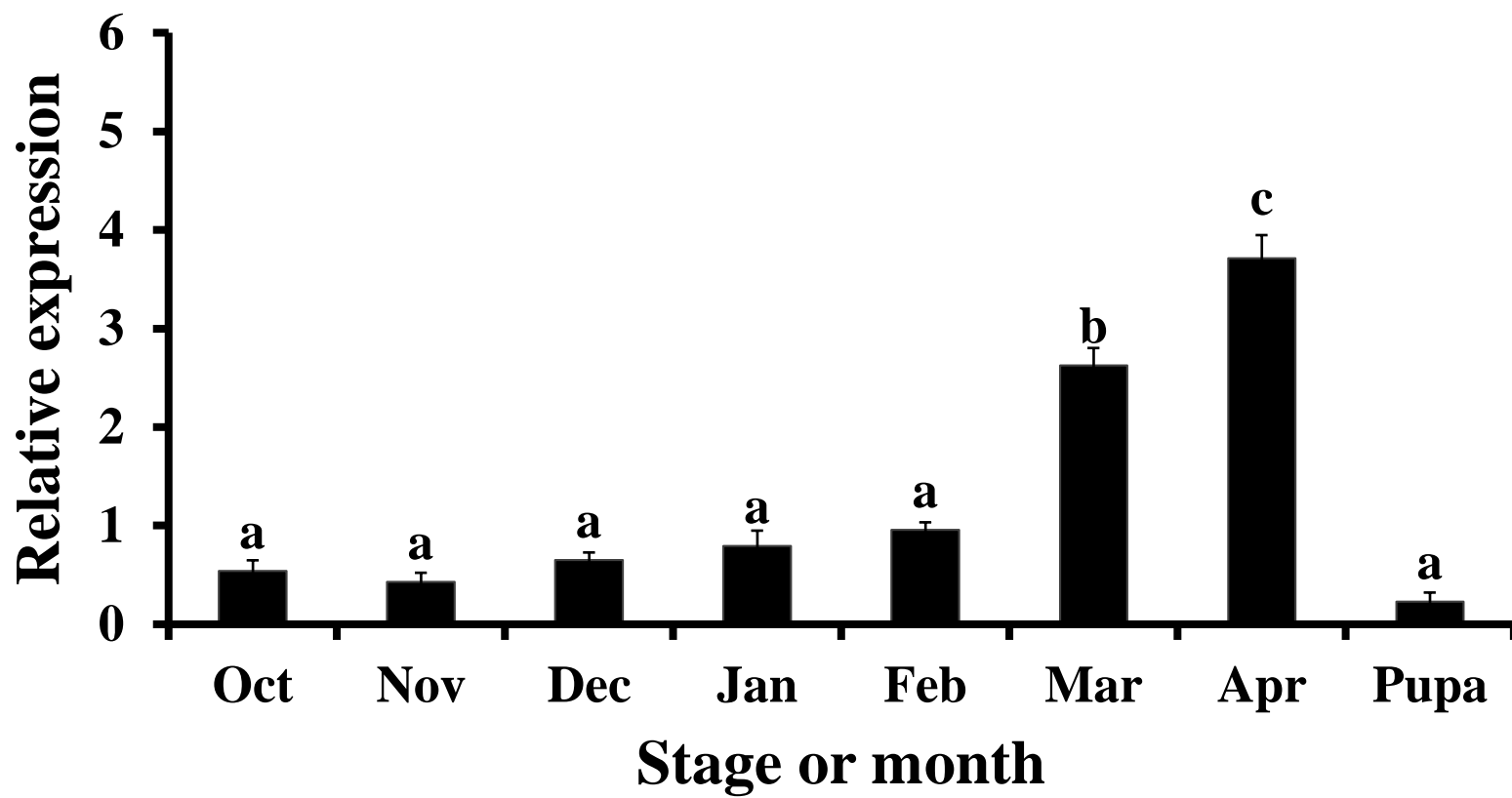

Supplement: S1 Fig — The results are expressed as the relative expression after normalization against endogenous ribosomal protein mRNA (OfRpL3). Expression is relative to the gene expression in diapausing larvae from October (assigned a value of 1). Each value is the mean ± SEM of three independent experiments. Means with different letters indicate a significantly difference (ANOVA: n = 3, P < 0.05) (PDF) [file pone.0174919.s001.pdf]
