# Supplementary material for: Larval diapause termination in the bamboo borer, Omphisa fuscidentalis
Source: PLoS One. 2017 Apr 3;12(4):e0174919. doi: 10.1371/journal.pone.0174919 (PMC5378396; doi:10.1371/journal.pone.0174919)
Supplement: S2 Fig — (A) PCR product of OfDH-PBAN mRNA by using primers OfDH-SPF and OfDH-SPR. M, marker for DNA molecular weight. (B) Nucleotide sequence and the deduced amino acid sequence of the PCR products. (PDF) [file pone.0174919.s002.pdf]

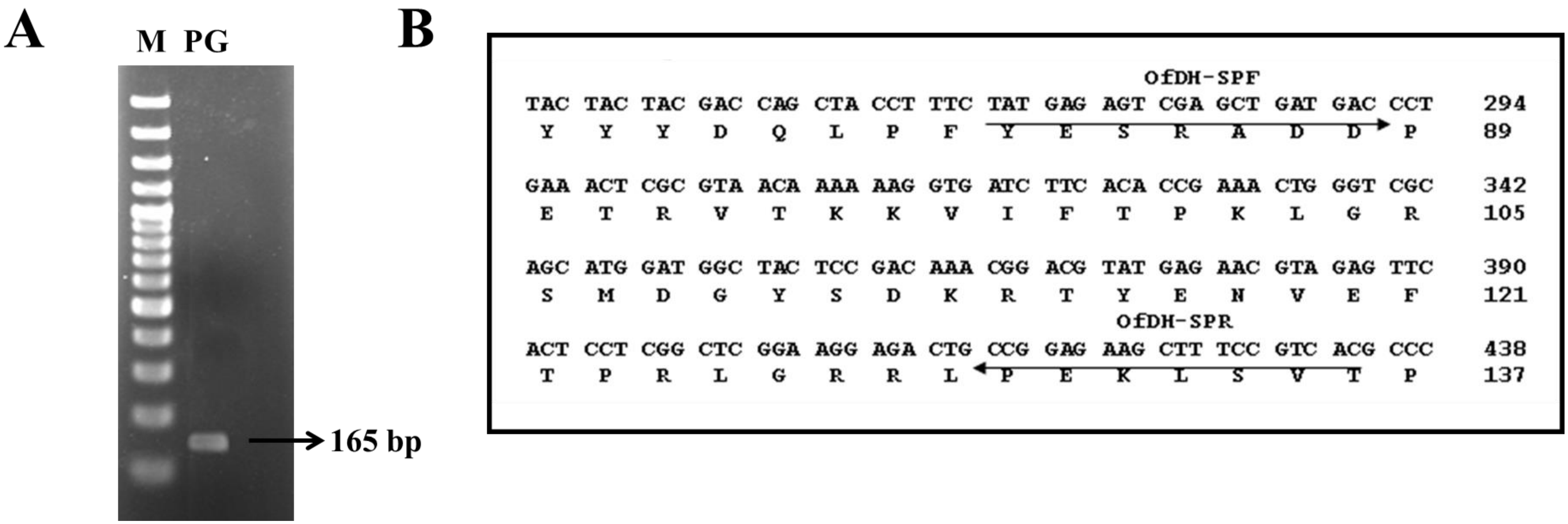
A
M PG
165 bp
B
OfDH-SPF
TAC TAC TAC GAC CAG CTA CCT TTC TAT GAG AGT CGA GCT GAT GAC CCT 294
Y Y Y D Q L P F Y E S R A D D P 89
GAA ACT CGC GTA ACA AAA AAG GTG ATC TTC ACA CCG AAA CTG GGT CGC 342
E T R V T K K V I F T P K L G R 105
AGC ATG GAT GGC TAC TCC GAC AAA CGG ACG TAT GAG AAC GTA GAG TTC 390
S M D G Y S D K R T Y E N V E F 121
OfDH-SPR
ACT CCT CGG CTC GGA AGG AGA CTG CCG GAG AAG CTT TCC GTC ACG CCC 438
T P R L G R R L P E K L S V T P 137
